# Effect of a Corset on the Gait of Healthy Beagle Dogs

**DOI:** 10.3390/ani11092650

**Published:** 2021-09-09

**Authors:** Takamasa Itoi, Shuji Kawata, Yoshiyuki Fukuda, Saori Maejima

**Affiliations:** 1 Faculty of Veterinary Medicine, Okayama University of Science, Imabari 794-8555, Ehime, Japan; 2 Department of Comparative Animal Science, Kurashiki University of Science and the Arts, Kurashiki 712-8505, Okayama, Japan; 3 Anifull, Division of Vet Supplies, Daiya Industry Co., Ltd., Okayama 701-0203, Okayama, Japan; kawata@daiyak.co.jp; 4 Department of R&D, Daiya Industry Co., Ltd., Okayama 701-0203, Okayama, Japan; fukuda@daiyak.co.jp; 5 Animal Rehabilitation Community, Okayama 701-1332, Okayama, Japan; maeji.kun.reha15@gmail.com

**Keywords:** gait analysis, dog, corset, rehabilitation, neurological disease, spinal cord

## Abstract

**Simple Summary:**

In recent years, corsets have been used in the prevention of nerve diseases in dogs and in their rehabilitation following surgery. The Anifull Dog’s Corset Pro, made by Daiya Industry Co., Ltd., is manufactured and sold for this purpose, but no studies have yet been conducted to verify its effectiveness. To evaluate the effects of the corset, we analyzed the gait of healthy beagle dogs that were or were not wearing the Anifull Dog’s Corset Pro. We found no difference in the walking speed of the dogs, but wearing the corset reduced the horizontal sway of the back. In conclusion, this corset does not affect the gait of dogs and may help body stability. Therefore, the Anifull Dog’s Corset Pro may be useful for the treatment of dog nerve conditions.

**Abstract:**

The prognosis for intervertebral disc disease (IVDD), a common neurologic disease in dogs, varies, with some cases requiring long-term rehabilitation. Corsets are used as part of the physical rehabilitation of dogs, and one of these, the Anifull Dog’s Corset Pro, is manufactured and sold by Daiya Industry Co., Ltd. This corset is used to relieve pain caused by spinal cord and vertebral diseases, and to prevent neurological conditions from worsening, by limiting spinal movement. However, the effect of the Anifull Dog’s Corset Pro on gait has not yet been clarified. Therefore, we aimed to evaluate the effects of this corset on the gait of dogs using kinematic and kinetic analyses. Five healthy beagle dogs wearing corsets were trotted, kinematic and kinetic parameters were measured using motion capture and force plates, and the results were compared to those obtained when the dogs were not wearing a corset. The range of motion of the angle formed by the 13th thoracic vertebra and the 7th lumbar vertebra at the apex of the 7th cervical vertebra was significantly reduced in the corset-wearing dogs. Thus, the Anifull Dog’s Corset Pro may improve trunk stability without affecting gait.

## 1. Introduction

Spinal cord injuries in dogs are principally caused by intervertebral disk disease (IVDD), traumatic lesions (e.g., fractures, luxation) [1,2,3] and vascular myelopathies (e.g., thromboembolism) [1,4,5]. IVDD is a common disease in dogs, and especially in chondrodystrophic breeds [6,7,8,9,10]. The severity of the spinal cord injuries caused by IVDD in the thoracolumbar region varies, but they can result in hyperesthesia in the chest, ataxia, paresis, and/or paraplegia of the hind limbs. The most important prognostic indicator of functional recovery is deep pain perception (DPP): surgical treatment is generally associated with a good prognosis if DPP is intact, but the recovery rate is only ~50% if DPP is lost [11,12,13,14,15,16]. Therefore, some dogs with IVDD require physical rehabilitation for a long period of time. In recent years, corsets have often been used as part of the rehabilitation process in many DPP negative dogs. In IVDD, degenerated discs impinge on nerve structures, which can cause pain and nerve dysfunction of varying severity. In addition, IVDD may be associated with dynamic compression. This degree of compression changes moment by moment according to the instantaneous position of the vertebral body due to movement. In other cases, stretching the fibers of the dorsal annulus or dorsal longitudinal ligament may cause pain by stimulating their nociceptors [8,17]. A number of corsets for dogs to manage pain care are on the market, one of which is the Anifull Dog’s Corset Pro made by Daiya Industry Co., Ltd. (Okayama, Japan). This corset is used to relieve pain caused by spinal cord injuries and diseases, including IVDD, and to prevent neurological conditions from worsening by restricting spinal movement. However, no previous studies have evaluated the effects of this corset in dogs. Gait analysis is necessary to fully evaluate its effectiveness.

Gait analysis includes kinematic and kinetic evaluations. Kinematic analysis assigns numerical values to motion by quantifying the position, velocity, acceleration, and angle of anatomical points, segments, and joints in space [18]. Kinetic analysis quantifies the forces involved by evaluating kinetic variables using a measurement system that includes a force plate. These kinetic variables include peak vertical and horizontal force values, vertical impulses, the strains in various tissues, loading rates, temporal gait characteristics, and the pressure distribution of the paw [18]. This type of computer-assisted gait analysis can be used to objectively evaluate various motion elements that cannot be perceived by the human eye. Kinematic and kinetic gait analyses have been used by many researchers [19,20,21,22,23].

The purpose of the present study was to evaluate the effect of the Anifull Dog’s Corset Pro on gait. For this purpose, kinematic and kinetic gait analyses of healthy beagle dogs were performed. We hypothesized that the Anifull Dog’s Corset Pro would stabilize the trunks of the dogs without changing their gait.

## 2. Materials and Methods

### 2.1. Ethics Approval

The experimental protocols used in this study were approved by the Kurashiki University of Science and the Arts Committee for Animal Experimentation (2018-03) and were performed in accordance with the National Institutes of Health Guidelines for the Care and Use of Laboratory Animals.

### 2.2. Animals and Experimental Design

Two healthy neutered male and three healthy intact female beagle dogs (mean ± SD 4.9 ± 0.7 years of age, body mass 12.3 ± 1.8 kg) were studied. None of the dogs had a history of lameness by orthopedic or neurological disease.

The dog corset (Anifull Dog’s Corset Pro) used in the study was made by Daiya Industry Co., Ltd. This corset is made of breathable mesh. Two 2-mm-thick dorsal resin plates are included to sandwich the spine and resist bending, and a 5-mm-thick cushion is included to improve the fit. The gait of the dogs was compared while trotting when wearing and not wearing a corset. Group A was defined as those who walked without a corset, and Group B was defined as those who walked with a corset.

### 2.3. Kinematic Data Acquisition

**Figure 1:**
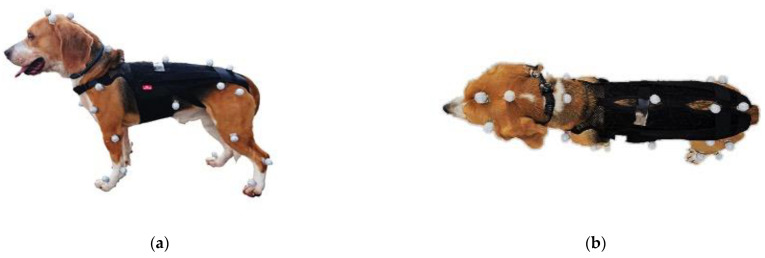
Reflective markers with a diameter of 8 mm were placed at 30 easily palpable anatomical landmarks to capture the gait of the dogs in three dimensions. (**a**) Sagittal plane; (**b**) Coronal plane. Data were collected for both (**a**) and (**b**) while the dogs were wearing and not wearing a corset.

**Figure 2:**
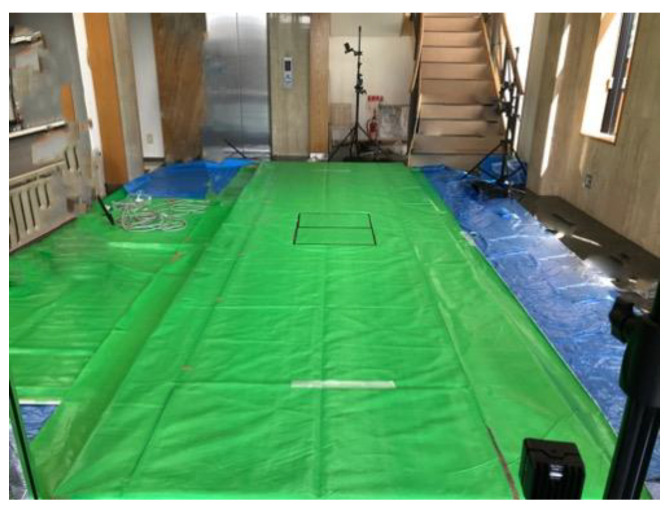
Walking path (5 m), which was surrounded by 12 thermal imaging cameras. Two force plates are placed in the middle of the walkway.

Reflective markers with a diameter of 8 mm were attached to various parts of the dog to capture their gait in three dimensions. A total of 30 markers were placed at clearly defined and easily palpable anatomical landmarks, as reported by Moore et al. [1]. These were the parietal region, the base of the left auricle, the atlanto-occipital joint, the seventh cervical vertebra, the thirteenth thoracic vertebra, the seventh lumbar vertebra, the tail crease, the iliac crest, the sternal pedicle, the tip of the last free rib, the middle of the scapula, the greater tubercle of the humerus, the lateral epicondyle of the humerus, the ulnar styloid process, the distal end of the fourth metacarpal, the greater trochanter of the femur, the lateral epicondyle of the femur, peroneal lateral malleolus, and the distal end of the fourth metatarsal (Figure 1). Regarding marker placement, it has been reported that skin movement can affect kinematic measurements [24]. To minimize soft tissue artifacts and make marker placement more reproducible, there is a method of placing markers on bony landmarks. However, this method is difficult to implement due to its high invasiveness in dogs. Furthermore, even if the markers are placed on bones, they are affected by soft tissue movement during walking. Therefore, in this study, the markers were placed on the skin. The positions of the markers were detected at 100 Hz by 12 infrared cameras (Prime 13 and Prime 13w, Natural Point Inc., Corvallis, OR, USA) surrounding the 5-m walkway and digitized using a calibrated three-dimensional space (Figure 2). Motive (Natural Point Inc., Corvallis, Oregon USA) was used for measurements and the data were analyzed using SKYCOM software (Acuity Inc., Tokyo, Japan).

### 2.4. Kinetic Data Acquisition

Two 400 mm × 600 mm (width × length) force plates (BP400600-2K, AMTI, Boston, MA, USA), adapted from 600 mm × 800 mm plates, were embedded in series within a straight 5-m walkway to measure the ground reaction forces for both thoracic and pelvic limbs during the same gait cycle (Figure 2).

### 2.5. Data Collection

A training phase of 5–10 min of walking was initially provided. The walking was performed with the same handlers, so that there would be no variation in gait. The data were considered to be valid when the dogs traveled along the walkway at a trot, in a straight line, without pulling to one side or turning their head, and placed two or more limbs on one force plate at the same time.

The walking speed of the dogs was measured. Additionally, the stance phase, swing phase, step length, stride length, and each joint angle (shoulder, elbow, carpal, hip, stifle, and tarsal joints) during one gait cycle were measured. Then, the ranges of motion (ROM) of the joints were calculated by subtracting the maximum extension angle from the maximum flexion angle, as indicators of the movements of the limbs. The stance phase percentage was calculated as stance time/gait cycle time × 100%. The swing phase percentage was calculated as swing time/gait cycle time × 100%. The step length was measured for each limb. The stride length was calculated as the distance between two consecutive ground contacts by the same limb. The angle of the back (the angle made between T13 and L7, with C7 as the apex in the coronal plane) during a single gait cycle was also measured. Then, the range of motion of the back was calculated using the maximum and minimum angles, as an index of trunk stability.

The force plates were used to measure the peak vertical force (PVF), vertical impulse (VI), peak braking force (PBF), peak propulsion force (PPF), and percentage weight-bearing on a limb when stationary. The PVF, VI, PBF, and PPF were normalized to the dog’s body mass (BM) and are quoted as %BM and %BM × s. All the data were collected during one gait cycle, but the dogs were trotted across the force plates until five valid datasets had been collected. Five sets of data were also collected with the dogs standing on the force plates. For each measurement, the mean values of the five repetitions were calculated.

### 2.6. Statistical Analysis

All the datasets were initially tested using the Shapiro-Wilk test, and then paired *t*-tests were performed when the data were normally distributed and Wilcoxon signed-rank tests were performed when they were not. The significance level was set at *p* < 0.05 and analyses were performed using SPSS version 25 (IBM, Inc., Armonk, NY, USA).

## 3. Results

The gaits of five healthy beagle dogs were analyzed when they were wearing and not wearing a corset.

### 3.1. Kinematic Analysis

#### 3.1.1. Walking Speed

The walking speed of the dogs while not wearing the corset was 2.02 ± 0.31 m/s and their walking speed while wearing the corset was 1.97 ± 0.27 m/s (Table 1). These were not significantly different.

#### 3.1.2. Movement of the Limbs

There were no differences in the ROM of each forelimb joint during one gait cycle when the dogs were wearing and not wearing a corset. The ROM of the left hip joint was significantly lower in the dogs when they were wearing a corset. But there were no differences in the ROMs of the other joints of the hind limbs. No effects of the corset were observed in dogs during the stance and swing phases of one gait cycle of trotting. There were also no obvious differences in step or stride length (Table 1).

#### 3.1.3. Trunk Stability

The ROM of the back in the coronal plane was 7.21 ± 3.82° in the dogs when they were not wearing a corset and 3.05 ± 1.21° when they were wearing a corset, which was significantly narrower (Table 1).

### 3.2. Kinetic Parameters

No obvious differences in PVF or VI in the extremities of the dogs were observed between the two conditions. Similarly, no significant differences in PBF or PPF were found between the two conditions. The weight-bearing rate of the forelimb at rest was 67.9% of BM when not wearing a corset and 69.78% of BM when wearing a corset. For the hind limbs, the weight-bearing rates were 32.1% of BM and 30.22% BM when the dogs were not wearing and wearing a corset, respectively. No significant differences in the weight bearing rates of the forelimb and hindlimb were found between the two conditions (Table 2).

## 4. Discussion

In the present study, we found no significant differences in specific kinematic and kinetic parameters between dogs when they were wearing or not wearing a corset, and the ROMs of their backs were significantly lower while wearing the support. Therefore, wearing a corset may stabilize the trunk of dogs without changing their gait.

Velocity is important in motion analysis and differences in velocity affect forces such as PVF. Kinematic parameters have been reported to be affected by the speed of motion and the joint ROM increases with increasing speed [25,26,27,28]. In the present study, all the dogs trotted at speeds of ~2.0 m/sec under each condition, probably because they were of the same breed and had similar body sizes. The lack of difference in speed under the two conditions implies that the conditions were similar when the dogs were and were not wearing a corset.

The ROM of the hip joint was significantly lower when the dogs were wearing a corset. Hip joint motion has a significant effect on the step and stride lengths. For instance, dogs with osteoarthritis of the hip joint owing to hip dysplasia have long strides and their PVF and VI are low [29,30,31]. However, the present study found no clear differences in kinematic parameters, including stride length, or in kinetic parameters, including PVF and VI, between the two conditions. Furthermore, when lameness is present in one limb, the other limbs compensate [32,33,34]. Bockstahler et al. [35] suggested the development of interarticular interactions and compensatory mechanisms because they found that dogs with radiographic evidence of hip dysplasia have larger flexion angles and ROMs of the femorotibial joints, and because the angular velocities during maximal flexion are significantly greater in the stifle and tarsal joints, in response to the limited ROM of the hip joint.

The findings of another study indicated that osteoarthritis of the hip joints leads to complex changes in gait pattern that involve joints other than the affected hip joints [22]. Thus, if the ROM of the hip joint is limited by the corset, it is possible that other joints will also be affected. Nevertheless, our results showed no significant differences in the joints in each of the extremities, except for the ROM of the left hip joint. Furthermore, the percentages of forelimb and hindlimb weight-bearing at rest were similar under the two conditions. These forelimb and hindlimb weight-bearing rates were similar to the weight-bearing rates of healthy dogs that were previously identified [36]. Therefore, despite the significantly lower ROM of the left hip joint in dogs wearing a corset, other joints were not affected, which suggests that the effect was small.

The corset incorporates two large, 2-mm-thick plastic plates in its long axis. The purpose of this corset is to relieve pain caused by the dynamic compression in IVDD and the stimulation of nociceptors by stretching fibers in the dorsal annulus or of the dorsal longitudinal ligament and to prevent the worsening of neurological symptoms [8,17]. Wearing this corset significantly reduced the ROM of the angle formed by the thirteenth thoracic vertebra and the seventh lumbar vertebra at the apex of the seventh cervical vertebra. In other words, the corset prevented the hips from moving from side to side during trotting, probably because the plastic plates sandwiched the vertebrae, thereby stabilizing the spine. This suggests the possibility of reducing back pain caused by IVDD and inhibiting the development of IVDD.

One previous study showed that dachshunds with thoracolumbar disc disease have more pronounced asymmetric trunk sway toward the side of the compression injury during walking. The authors speculated that this is the result of a loss of control of the hind limb on the side of the compression injury, which makes it easier for the animal to fall toward the affected side [37]. Another study that used kinematic and kinetic analyses to determine the effect of treatment on cervical spondylosis in dogs showed that trunk sway decreased with the improvement in gait following treatment [38]. These results indicate that the control of trunk sway improves the gait of dogs with spinal cord injury. The Anifull Dog’s Corset Pro used in the present study reduced the ROM in the back in the horizontal plane, which implies that it would support dogs with limb paresis while in motion. Further study is needed, but this suggests that it may be possible to ground the limb more correctly in dogs with spinal cord injury. In a previous study of treadmill training in rats with spinal cord injury, it was shown that forced plantar ground contact improves neurological function and gait, as well as the histology, including axonal density [39]. This implies that wearing a corset may contribute not only to the prevention and relief of hypersensitivity associated with IVDD, but also to the treatment of spinal cord injury.

In the past, corsets have been used to replace lost function. However, in recent years, it has been reported that rehabilitation can have an additive or synergistic effect to surgical treatment and regenerative medicine in the treatment of spinal cord injury [40,41,42,43,44]. Thus, the existence of a therapeutic aspect to rehabilitation is beginning to be more widely recognized in veterinary medicine, and it may be useful for dogs that have not been able to regain normal gait following surgical treatment. There are many methods of rehabilitation. In IVDD, it is essential to ensure spinal stability in both postoperative and conservative treatment. Therefore, wearing the Anifull Dog’s Corset Pro before surgery may allow for early treatment and rehabilitation.

To date, we have not found any report in the Medical and Biological Literature Database (PubMed: https://pubmed.ncbi.nlm.nih.gov, accessed on 1 May 2021) examining the effects of corsets on healthy dogs using kinematic and kinetic analysis. Therefore, this is the first study to analyze the effects of canine corsets in healthy dogs using kinematic and kinetic walking analysis. By studying a single breed, we were able to eliminate the effects of breed-specific differences in anatomy. However, this study had some limitations. First, a single breed of healthy dogs were used in the present study. Therefore, the results obtained in this study do not indicate efficacy for treatment, but rather the possibility of contributing to gait stability without the occurrence of adverse events. In order to validate the usefulness of corsets, it is necessary to study its use in multiple breeds of dogs with neurological diseases. Second, the sample size was small. A larger sample would have reduced the influence of data collected from a single dog and minimized differences between and within individuals. Third, there was the possibility of soft tissue artifacts, which are a common source of error in human and equine gait analysis. In particular, it has been reported that skin movements can affect kinematic measurements [24]. To minimize soft tissue artifacts and make marker placement more reproducible, markers could be placed on bony landmarks, but this is associated with ethical issues. Fourth, the co-authors involved in this study include people who work for the company that makes this product. This may not necessarily eliminate all biases in the results and interpretation of this study. Finally, the study period for each dog was very brief, a matter of minutes. Long-term usage of these corsets may have very different effects. Furthermore, because the effect of the corset was not evaluated in every joint, it will be necessary to conduct investigations over a longer period of time and evaluate joint moments evaluated in the future.

## 5. Conclusions

This is a preliminary study with a small sample size and the limited conclusions that can be drawn from the use of a single breed of healthy dogs. However, we have analyzed the effects of the Anifull Dog’s Corset Pro on the gait of dogs and found that it reduced the ROM of the left hip joint, but that this did not have a significant effect on gait. Moreover, the weight-bearing rates of the forelimb and hindlimb in this study were similar to those in a previous study. Furthermore, the ROM of the back in the horizontal plane was lower, which suggests that it stabilizes the trunk. Thus, the Anifull Dog’s Corset Pro may represent a useful tool for the postoperative rehabilitation of IVDD and spinal motion in the thoracolumbar region and related pain, and may also help prevent their development. In the future, it will be necessary to study dogs with such conditions and to study the joint moments, to evaluate the effects of the corset on each joint.

## Figures and Tables

**Table 1:** Kinematic parameters in dogs while wearing and not wearing a corset.

Kinematic Variable (Mean ± SD)	A		B		*p*-Value A vs. B	
	Left Forelimb	Left Hindlimb	Left Forelimb	Left Hindlimb	Left Forelimb	Left Hindlimb
	Right Forelimb	Right Forelimb	Right Forelimb	Right Forelimb	Right Forelimb	Right Forelimb
Walking speed (m/s)	2.02 ± 0.31		1.97 ± 0.27		0.65	
stance phase (%)	43.78 ± 5.04	32.46 ± 2.10	44.02 ± 4.22	34.37 ± 2.47	0.91	0.15
	44.65 ± 4.67	32.63 ± 1.48	45.85 ± 4.23	33.68 ± 3.12	0.47	0.51
swing phase (%)	56.23 ± 5.04	67.54 ± 2.10	55.98 ± 4.22	65.63 ± 2.47	0.91	0.15
	55.35 ± 4.67	67.37 ± 1.48	54.15 ± 4.23	66.32 ± 3.12	0.47	0.51
step length (cm)	38.68 ± 3.78	38.61 ± 4.09	35.99 ± 2.86	35.35 ± 3.15	0.16	0.10
	38.53 ± 2.94	38.16 ± 2.25	36.90 ± 2.83	37.83 ± 3.04	0.19	0.72
stride length (cm)	77.38 ± 5.47	77.52 ± 4.68	73.59 ± 3.24	73.00 ± 3.59	0.13	0.13
	77.82 ± 5.84	77.28 ± 5.63	73.68 ± 3.48	73.78 ± 4.03	0.19	0.27
shoulder joint (°)	26.27 ± 2.69		30.32 ± 5.97		Left: 0.24	Right: 0.21
	40.00 ± 7.38		35.90 ± 3.45			
elbow joint (°)	68.20 ± 6.20		69.07 ± 6.18		Left: 0.71	Right: 0.52
	77.31 ± 8.19		75.88 ± 10.01			
carpal joint (°)	122.49 ± 19.34		115.17 ± 16.68		Left: 0.12	Right: 0.43
	127.37 ± 20.49		123.86 ± 15.49			
hip joint (°)		30.12 ± 4.82		24.52 ± 6.61	Left: 0.02 *	Right: 0.48
		32.97 ± 5.49		31.10 ± 4.41		
stifle joint (°)		63.93 ± 9.97		66.05 ± 9.85	Left: 0.31	Right: 0.61
		66.51 ± 11.46		68.04 ± 10.14		
tarsal joint (°)		57.26 ± 3.73		59.61 ± 6.76	Left: 0.43	Right: 0.27
		64.41 ± 7.39		60.26 ± 8.53		
back (°)	7.21 ± 3.82		3.05 ± 1.21		0.03 *	

Data are mean ± SD. * *p* < 0.05 for corset-wearing vs. non-corset-wearing; paired *t*-test. A: non-corset-wearing, B: corset-wearing.

**Table 2:** Kinetic parameters measured in the dogs while wearing and not wearing a corset.

Kinetic Variable (Mean ± SD)	A		B		*p*-Value A vs. B	
	Left Forelimb	Left Hindlimb	Left Forelimb	Left Hindlimb	Left Forelimb	Left Hindlimb
	Right Forelimb	Right Forelimb	Right Forelimb	Right Forelimb	Right Forelimb	Right Forelimb
PVF (% BW)	121.45 ± 13.43	88.43 ± 5.10	125.32 ± 16.63	84.21 ± 7.40	0.59	0.13
	117.90 ± 17.91	86.19 ± 6.40	121.72 ± 14.74	83.54 ± 8.91	0.46	0.53
VI (% BW × s)	11.39 ± 2.04	6.80 ± 0.97	12.07 ± 1.39	6.41 ± 1.10	0.13	0.35
	11.35 ± 1.94	6.81 ± 1.07	11.65 ± 1.38	6.42 ± 1.07	0.46	0.08
PBF (% BW)	18.89 ± 5.08	4.63 ± 3.47	17.83 ± 5.37	4.51 ± 1.82	0.53	0.93
	16.03 ± 3.76	4.68 ± 2.30	17.55 ± 5.31	4.82 ± 1.87	0.38	0.93
PPF (% BW)	8.71 ± 2.69	13.12 ± 2.61	7.8 ± 2.34	11.74 ± 3.67	0.56	0.25
	8.62 ± 2.29	12.23 ± 2.62	7.12 ± 1.42	10.21 ± 1.81	0.25	0.05
weight bearing (% BW)	forelimb 67.9 ± 0.05	hindlimb 32.1 ± 0.05	forelimb 69.78 ± 0.03	hindlimb 30.22 ± 0.03	forelimb: 0.34	hindlimb: 0.34

Data are mean ± SD. PVF: peak vertical force, VI: vertical impulse, PBF: peak braking force, PPF: peak propulsion force.

## Data Availability

Not applicable.
